# Dr. C. G. Davis

**Published:** 1852-01

**Authors:** 


*Dr. C. G. Davis,* a graduate of the Baltimore College of Dental Surgery, and formerly of Sippican, Mass., has recently removed to New Bedford, Mass., a place which opens to him a larger field for the exercise of his fine professional abilities. Dr. D. is devotedly attached to his profession, and urged on by an ambition that will not permit him to rest satisfied with mediocrity of attainment, we feel assured his merits as a practitioner will not remain long unappreciated by the citizens of the place of which he has now become a resident.

